# Diapause-Associated Protein3 Functions as Cu/Zn Superoxide Dismutase in the Chinese Oak Silkworm (*Antheraea pernyi*)

**DOI:** 10.1371/journal.pone.0090435

**Published:** 2014-03-10

**Authors:** Zhenle Bi, Xiaoli Yang, Wei Yu, Jianhong Shu, Yaozhou Zhang

**Affiliations:** 1 College of Life Sciences, Zhejiang Sci-Tech University, Hangzhou, China; 2 Tianjin international Joint Academy of Biomedicine, Tianjin, China; University of Arkansas for Medical Sciences; College of Pharmacy, United States of America

## Abstract

To better understand the molecular mechanism underlying of diapause in *Antheraea pernyi* (*A.pernyi*), we cloned a novel diapause-associated protein 3 (DAP3) gene from *A.pernyi* by reverse transcription-polymerase chain reaction (RT-PCR) and studied the biological functions. Sequence analysis revealed that this gene encodes 171 amino acids and has a conserved domain of Copper/Zinc superoxide dismutase (Cu/Zn-SOD). Western blot and qRT-PCR results showed that DAP3 was mainly expressed in the pupal stage, and gradually decreased as diapause development. DAP3 was also expressed in 1st and 5th instar larvae of *A.pernyi*. In tissues of 5th instar larvae of *A.pernyi*, DAP3 was mainly expressed in the epidermis, followed by the head, hemolymph and fat body. To identify the SOD activity of DAP3, we constructed a prokaryotic expression vector by inserting the coding region sequence into plasmid pET-28a (+) and obtained the purified rHIS-DAP3 fusion protein by Ni-NTA affinitive column. Importantly, we found the SOD activity of DAP3 fusion protein was approximately 0.6674 U/µg. To further confirm the SOD activity of DAP3 in vivo, we induced the oxidative stress model of pupae by UV irradiation. The results showed that both the mRNA and protein level of DAP3 significantly increased by UV irradiation. Furthermore, the SOD activity of the total lysate of pupae increased significantly at 10 min post UV irradiation and transiently returned to normal level afterwards. These results suggested that DAP3 might be a novel protein with SOD activity getting involved in regulation of diapause in *A.pernyi*.

## Introduction

Diapause is a specific physiological phenomenon in living organisms, in which growth and activity are temporarily suspended in the developmental stage. Many insects make effective use of diapause in their life cycles to overcome unfavorable seasons [1]. *A.pernyi* is an insect that survive the winter season by pupal diapause. However, the detailed molecular mechanisms underlying the diapause of *A.pernyi* remain unknown. Diapause-associated protein (DAP) is abundant in the fat body or hemolymph in diapause insects; meanwhile, little or no DAP can be found in non-diapause insects [2], [3].This protein was first found in *Lepanotarsa dacemlineata* and then later studied in *Diatraea grandiosella*, *Buesseola fusca*, *Gastrophysa atrocyanea* and *Pectinophora gossypidlla* [4]. It is generally believed that hormone change in the insect is closely related to the occurrence of diapause [5]. When hormones are suitable for DAP generation, diapause occurs, otherwise, diapause is terminated [5].

Reactive oxygen species (ROS) generated from metabolic processes attack organic macromolecules, including proteins, nucleic acids and membrane lipids. The oxidative stress not only causes injuries and pathological deterioration, but also leads to many physiological events correlated with cancer, mutagenesis, cell death, degenerative processes and aging [6]. The superoxide diamutase (SOD) is an antioxidant enzyme that commonly found in aerobic organisms for removing superoxide anion free radical (O_2_^.−^) and forms the first line of defense against ROS. SOD catalyzes the diamutation of O_2_^.−^∶2O_2_^.−^+2H^+^→H_2_O_2_+O_2_ [7], [8] and plays an important role for the dynamic equilibrium of generation and elimination of O_2_^.−^ [9]. SOD was divided into three kinds of iron-SOD (Fe-SOD), manganese-SOD (Mn-SOD) and copper/zinc-SOD (Cu/Zn-SOD) basing on metal requirements of the active sites [10]. Cu/Zn-SOD is a well-studied protein, found primarily in the cytosol of eukaryotes and some kinds of bacteria and fungi, which plays critical roles in immune response [10], [11]. Cu/Zn-SOD is bound to one copper and one zinc ion and displays the Greek key beta-barrel fold [12]. The Cu/Zn-SOD molecule consists of two subunits and forms dimer with the assistance of hydrophobic and hydrogen bond. Within the peptide chain, the disulfide bridge, which is constituted of C55 and C144 cysteine sulfydryl, also plays an important role in subunit association process.

Studies of time interval measuring enzyme-esterase A4 (TIME-EA4) from diapause eggs of the silkworm *Bombyx mori (B.mori)* show that the protein is a time-dependent ATPase that may regulate the diapause duration of *B.mori* eggs [13]. TIME-EA4, which also named diapause bio-clock protein or TIME, had been identified as an esterase, acting as a single, transitory activation burst for the termination of the *B.mori* diapause two weeks after eggs had been chilled at 5°C [14]. The possible timer function may arise from a built-in mechanism in the protein structure of TIME-EA4 [14], [15], [16]. Peptidyl inhibitory needle (PIN), which was identified as a time measurement-regulating peptide, binds with TIME-EA4 protein to inhibit the activation of ATPase and consequently to regulate time measurement by TIME-EA4 [14], [15], [16].The amino acid sequence of TIME-EA4 shows 46% to 55% homology with the proteins of Cu/Zn-SOD family. The timer function is not in the SOD core domain and TIME-EA4 has an attached sugar chain, which is indispensable to its functioning as a timer protein [17], [18].

In this study, we identified DAP3 (GenBank login number: AFC35302.1) as a novel Cu/Zn-SOD protein, which might play potential roles in regulation of *A.pernyi* diapause.

## Materials and Methods

### Materials and main reagents

*E.coli* TG1, BL21 (DE3) is kept in our laboratory. TRIzol Reagent was purchased from Ambion Company (USA). HT Superoxide Dismutase Assay Kit were products of TREVIGEN Company (USA). SYBR Green I and DNase I were purchased from Roche Company (USA).

### Transcription spectrum analysis of the *DAP3* gene

We used TRIzol reagent to isolate the total RNA of different developmental stages insects including diapause pupae, non-diapause pupae, pupae in the period of diapause development for different days, moth, eggs, developed eggs and 1st to 5th instar larvae of *A.pernyi* and different tissues of 5th instar larvae of *A.pernyi* including epidermis, ovary, fat body, hemolymph,midgut,malpighian tubule, trachea, silkgland.After digested by DNaseI for 30 min at 37°C,the RNA was reverse transcribed into cDNA according to the protocol for RevertAid First Strand cDNA Synthesis Kit (Thermo Scientific, USA). We designed two pairs of primer to amplify *DAP3 gene* (Forward: 5′-GAAGGCAGTATCGTCGGTCTA-3′; Reverse: 5′-TGTGTTCTGGGTTGAAATGAGC-3′) and*β-actin* gene (Forward: 5′-ACCAACTGGGACGACATGGAGAAA-3′; Reverse: 5′-TCTCTCTGTTGGCCTTTGGGTTGA-3′), respectively. The reaction system was as follows: 1 µL of cDNA template, 0.5 µM forward/reverse primer, 10 µL of 2×SYBR Green I Master and 7 µL of PCR grade water. The result was detected in the Light Cycler 480 with three repeats for each reaction.

### Western blot analyses

Total proteins of the *A.pernyi* insects were extracted and the protein concentration was determined by BCA method [19]. After that, the aliquots of 50 µg samples on each lane were separeted by 12% SDS-PAGE and transferred to the polyvinydene fluoride (PVDF) membrane at 4°C for 2 h at 150 mA. Subsequently, the membrane was blocked in 5% bovine serum albumin (BSA) for 2 h, followed by incubation in the DAP3 antibody diluent (1∶5000) for 1 h and Goat anti-mouse IgG (H+L)-HRP diluent (1∶10000) for 30 min after washed for three times with Tris-Buffered Salinewith Tween 20 (TBST: 50 mM Tris, 150 mM NaCl, 0.05%Tween 20, pH 7.6). Finally, the protein bands were visualized using the Enhanced Chemiluminescence kit (Pierce, USA) and β-actin was used as loading controls for normalizing band intensity.

### PCR amplification of the DAP3 gene

Total RNA was isolated from 50 mg fat body of *A. pernyi* by TRIzol Reagent and the RNA integrity was detected with 1% TBE agarose gel electrophoresis. The cDNA fragment was generated using RevertAid First Strand cDNA Synthesis Kit following the manufacturer's potocol. We designed primers for PCR to obtain the open reading frame (ORF) of the *DAP3* gene:DAP3-F: 5′-GGGGATCCATGCAACCGACACG-3′ and DAP3-R:5′-GAAGCTTTTACAAAATTCCGATAACACCAC. And the specific PCR procedures is as follows: initial denaturation at 94°C for 5 min; followed by 35 cycles each denaturation at 94°C for 30 s, annealing temperature at 57°C for 30 s, extension at 68°C for 40 s; with final extension at 68°C for additional 7 min.

### Construction of bacterial expression vector and sequence analysis

The amplified fragment (492 bp) after digestion with *Bam*H I and *Hind* III was inserted into pET-28a(+) vector digested with the same restriction enzymes by Ligation High.The production was transferred into *E.coli* strain TG1 to screen the postive clone. And the positive recombinant plasmid was confirmed by sequencing.

### Expression and purification of DAP3 recombinant protein and SOD activity assay

The recombinant plasmid was transformed into *E.coli* strain BL21. A single positive colony was incubated in Luria-Bertani (LB) medium with 40 mg/L Kanamycin at 37°C untill the value of OD_600_ reached 0.5–0.7 and isopropylthio-β-D-galactoside (IPTG) was added to a final concentration of 1 mM for fusion protein expression. After inducing, the pelleted cells were harvested by centrifugation for 20 min at 6,000 rpm and re-suspended in PBS buffer. Subsequently, the cells were homogenized by ultrasonic machine and the fusion protein was purificated by Ni-NTA agarose. The SOD activity of the purified recombinant DAP3 was determined using HT Superoxide Dismutase Assay Kit. In this assay, O_2_^.−^, generated from the conversion of xanthine to uric acid and hydrogen peroxide (H_2_O_2_) by xanthine oxidase (XOD), convertsthe tetrazolium salt WST-1 to WST-1 formazan, which absorbs light at 450 nm. The SOD reduces O_2_^.−^concentrations and thereby lower the rate of WST-1 formazan formation. Absorbance is measured at 450 nm using a standard spectrophotometer.

### Oxidative stress response assay

Fresh pupae were divided into four groups: group1was control group with its pupae were not irradiated by UV; group2, 3 and 4 were exposed to UV light for 10, 20 and 30 min, respectively. Each group had 3 pupae and the ultraviolet irradiance is 300 µW/cm^2^. After oxidative stress, the pupae were sacrificed for qRT-PCR and Western blot detection. The SOD activity of the total protein in each group was also analyzed.

### Data analysis and statistics

The Western blot results were quantified using ImageJ (NIH) and normalized with β-actin as loading controls. Quantitative data (mean ± SEM) were from at least 3 independent experiments. For significant analysis, oneway ANOVA was performed using Prism 5 (Graph Pad) software (*p<0.05 and **p<0.01 were considered significant difference).

## Results

### Sequence analysis of DAP3

**Figure 1:**
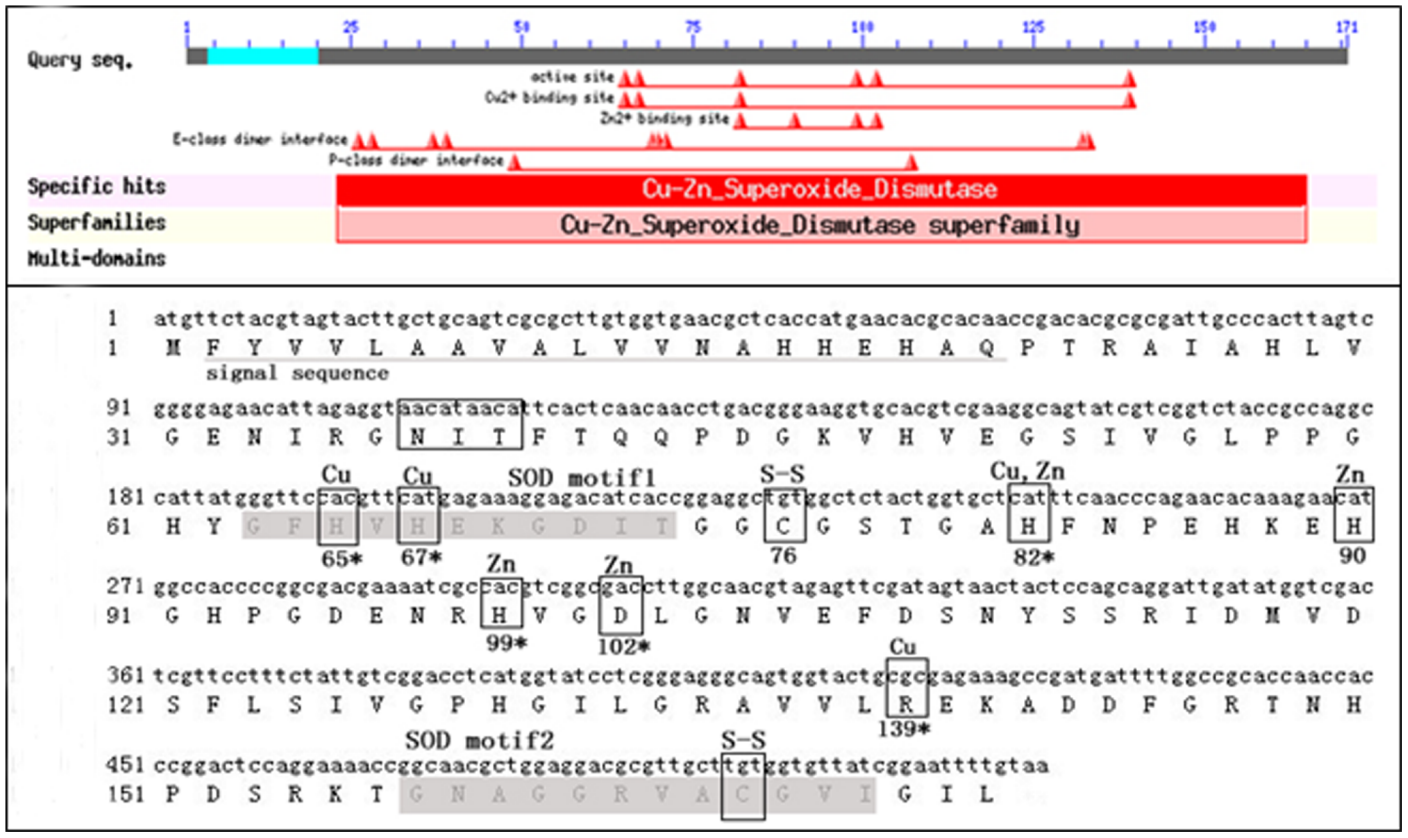
Amino acid sequence analysis of DAP3. (**a**) The conserved SOD domain of DAP3 revealed by BLASTp analysis online (http://www.ncbi.nlm.nih.gov/BLASTp). (**b**) Specific motifs of DAP3 protein. NIT in the box is the glycosylation site, the shading characters are SOD domain and the asterisks indicate amino acids of oxidative modification sites. Cu = copper binding, Zn = zinc binding, Cu, Zn = copper and zinc binding, S-S = disulfide bond.

Sequence analysis showed that *A.pernyi* DAP3 has a full length of 572 nucleotide acids encoding a putative protein of 171 amino acid residues. The predicted molecular weight is 18.19 kDa and isoelectric point is 6.6. Importantly, an active SOD motif (SOD motif 1 and 2) is found in the protein. As shown in Figure 1, the SOD domain includes metal ligand amino acid residues (His 65, His 67, His 82 and Arg 139 for copper ion and His 82, His 90, His 99 and Asp 102 for zinc ion) and a disulfide bond (between Cys 76 and Cys 166), which are highly conserved in the SOD protein family.

**Figure 2:**
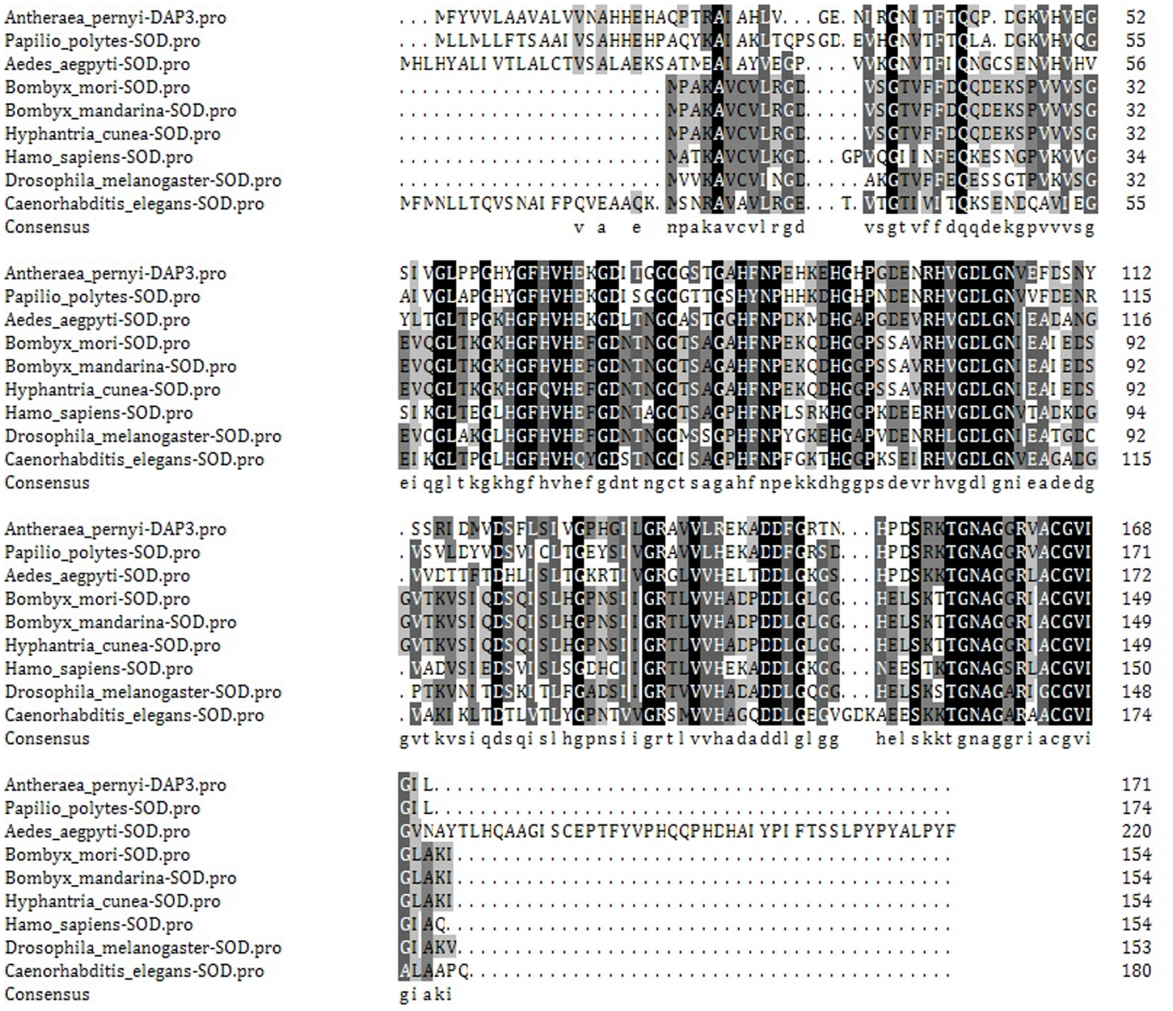
Alignment of amino acid sequences of DAP3 and Cu/Zn-SODs. The amino acid sequence alignment of *A.pernyi* DAP3 with *Papilio polytes*, *Aedesa egpyti*, *Bombyx mori*, *Bombyx mandarina*, *Hyphantria cunea*, *Homo sapiens*, *Drosophila melanogaster* and *Caenorhabditis elegans* Cu/Zn-SOD.

As shown in Figure 2, the amino acid sequence of *A.pernyi* DAP3 was aligned with Cu/Zn-SOD of *Papilio polytes* (BAM19071), *Aedes aegpyti* (XP004535893), *Bombyx mori* (AB179561), *Bombyx mandarina* (EF077625), *Hyphantria cunea* (AB290453), *Homo sapiens* (NM_000454), *Drosophila melanogaster* (M24421) and *Caenorhabditis elegans* (P34697). The homology percent was 69%, 49%, 48%, 48%, 47.3%, 52.3%, 46.6% and 41.3%, respectively.

**Figure 3:**
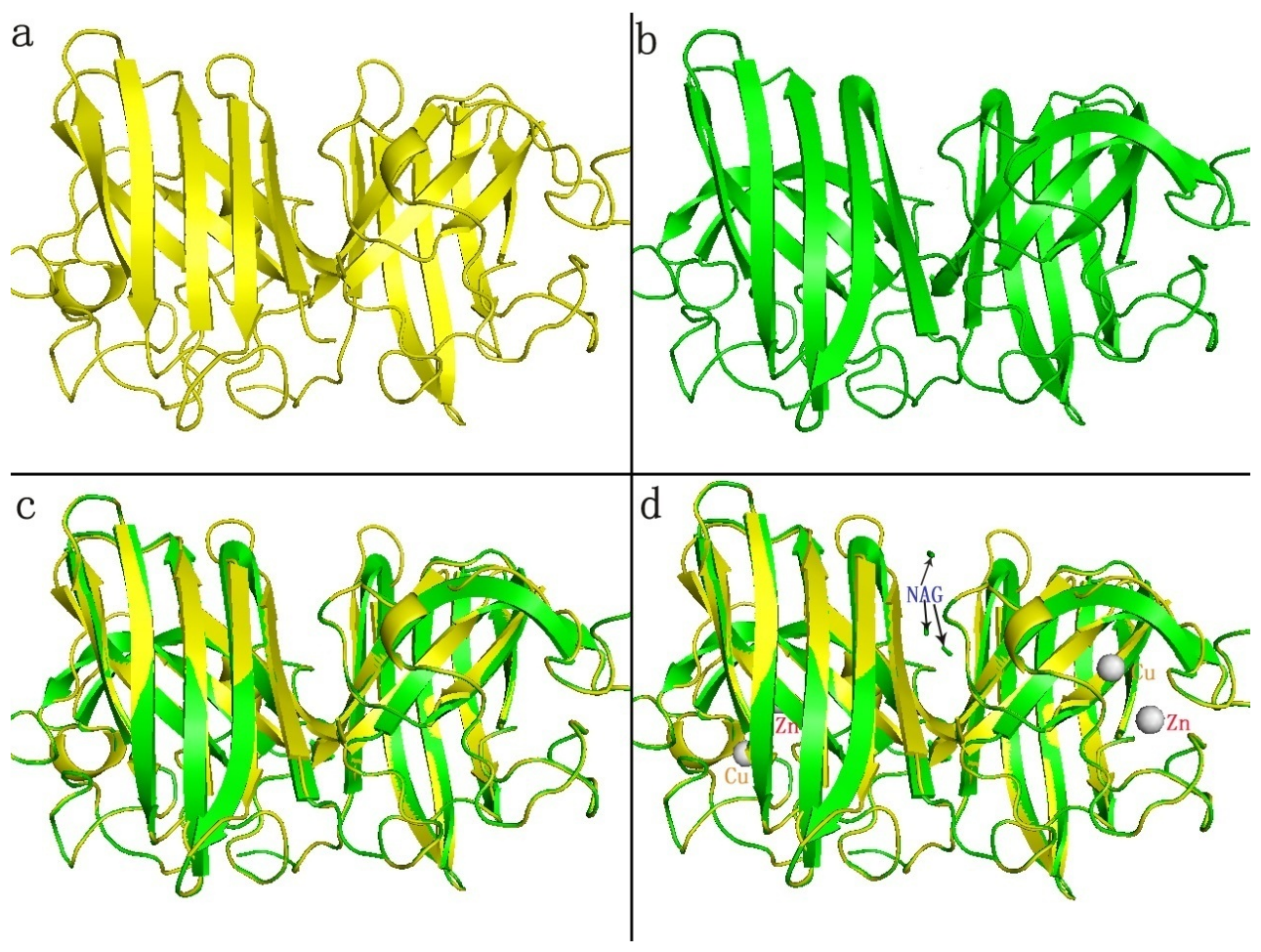
The putative 3D structure of *A.pernyi* DAP3 and alignment with *B.mori* TIME-EA4. (**a**) The putative tertiary structure of *A.pernyi* DAP3. (**b**) Tertiary structure of *B.mori* TIME-EA4. (**c**) The alignment of *A.pernyi* DAP3 with *B.mori* TIME-EA4. (**d**) The 3D structure with metal ions and glycosylation modification.

SWISS-MODEL (http://swissmodel.expasy.org/) predicted the 3D structure of *A. pernyi* DAP3 online. Furthermore, we used software PyMOL to align *A.pernyi* DAP3 with *B.mori* TIME-EA4. As shown in Figure 3, among 305 aligned atoms, 7 atoms were rejected during cycle 1(RMS = 0.09) and 5 atoms were rejected during cycle 2(RMS = 0.08). The final RMS is 0.077 nm (293 to 293 atoms), indicating high homology existed in the two proteins.

### Expression profile of DAP3 in *A.pernyi*

**Figure 4:**
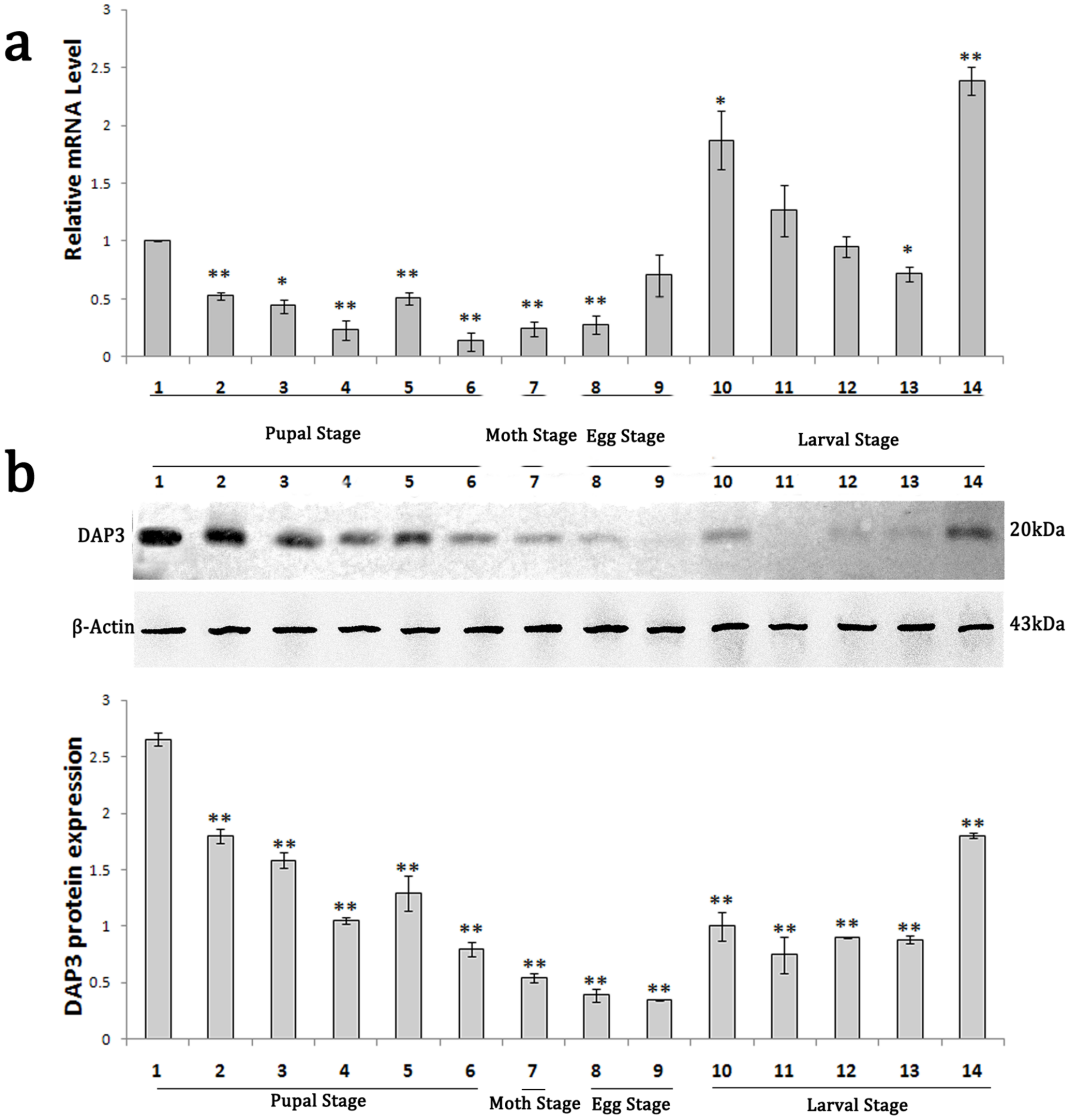
Transcription spectrum and Expression profile analysis of *A.pernyi DAP3* gene. (**a**) qRT-PCR of transcription level of *DAP3* in different developmental stages. 1: diapause pupae; 2: non-diapause pupae; 3–6: pupae in the period of diapause development for 4days, 8days, 11days and 14days; 7: moth; 8: egg; 9: developed egg; 10–14: 1st to 5th instar larvae *of A.pernyi*. Date represent mean ± SEM from three independent experiments. *p<0.01 vs diapause pupaereference, **p<0.01 vs diapause pupae reference. (**b**) Western blot of expression level of DAP3 in different developmental stages. Lane 1: diapause pupae; lane 2: non-diapause pupae; lane 3–6: pupae in the period of diapause development for 4days, 8days, 11days and 14days; lane 7: moth; lane 8: egg; lane 9: developed egg; lane 10–14: 1st to 5th instar larvae of *A.pernyi*. **p<0.01 vs diapause pupae.

The qRT-PCR results showed that *DAP*3 mRNA is highly expressed in the larvae (lane 10–14 in Figure 4a, fold change compared to 1), diapause pupae (lane 1) and developed eggs (lane 9) whereas the mRNA level is quite low in the non-diapause pupae (lane 2) and the pupae at the period of diapause development (lane 3–6). While the DAP3 protein expression level is high in the pupal stage (lane 1–6 in Figure 4b), it gradually decreased as diapause development (lane 3–6). Besides, in 1st and 5th instar larvae of *A.pernyi*, we also detected the protein expression of DAP3 by Western blot (Figure 4b).

**Figure 5:**
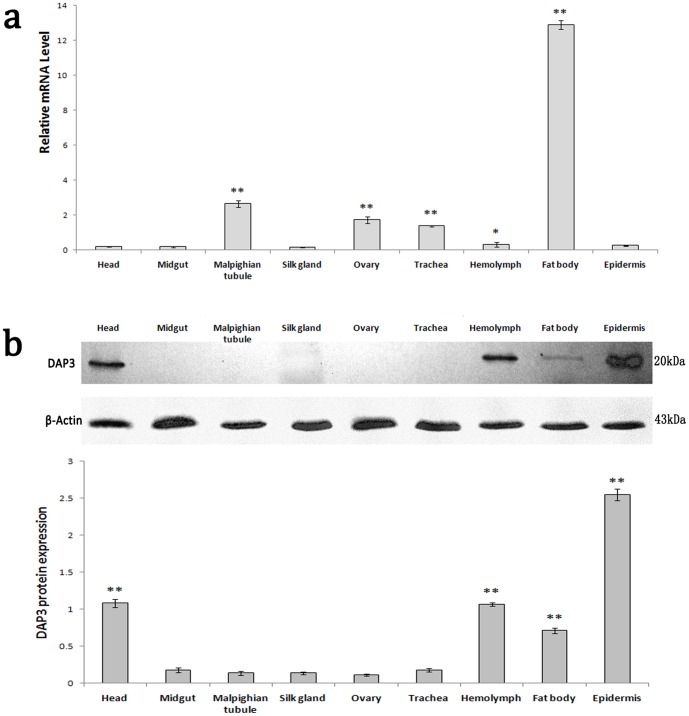
Transcription spectrum and Expression profile analysis of *DAP3* gene of *A.pernyi* 5th instar larvae. (**a**) qRT-PCR of transcription level of *DAP*3 in different tissues of 5th instar larvae *of A.pernyi* with β-actin as an internal control. Results are represented as mean ± SEM of three independent experiments. *p<0.05 vs midgut value, **p<0.01 vs midgut value. (**b**) Western blot of expression level of DAP3 in different tissues of 5th instar larvae *of A.pernyi*. **p<0.01 vs midgut value.

To identify the spatial distribution of DAP3 expression, the tissues of 5th instar larvae of *A.pernyi* were collected and examinated. In the different tissues of 5th instar larvae *of A.pernyi*, the highest level of transcription was found in the fat body, followed by malpighian tubule, ovary and trachea with very low level in other tissues (Figure 5a). DAP3 protein expression is highest in the epidermis, followed by the head,hemolymphand fat body, while no expression is found in the other tissues (Figure 5b).

### Production of DAP3 recombinant protein

**Figure 6:**
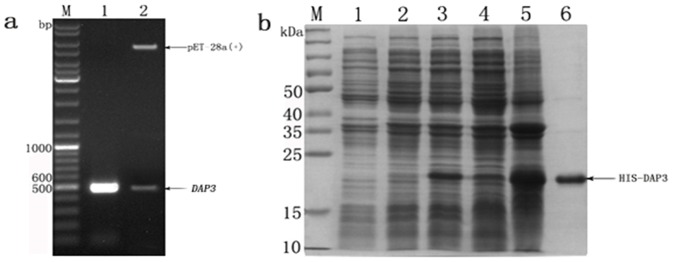
Construction of recombinant plasmid and production of DAP3 recombinant protein. (**a**) TAE agarose gel electrophoresis of PCR and double digestion analysis. Lane M:Gene Ruler 100 bp plus DNA Ladder; lane 1: PCR with the recombinant plasmid as template; lane 2: Digestion with *Bam*H I and *Hind* III. (**b**) SDS-PAGE analysis of DAP3 recombinant protein in *E.coli* BL21 cells. Lane M: protein moleculai mass marker; lane 1: *E.coli* BL21 containing expression vector pET28a(+) induced by IPTG; lane 2: *E.coli* BL21 cells containing expression vector pET28a(+)-*DAP*3 without IPTG; lane 3: *E.coli* BL21 cells containing expression vector pET28a(+)-*DAP*3 induced by IPTG; lane 4: Supernatant solution after ultrasonic; lane 5: Precipitation after ultrasonic; lane 6: purified protein by Ni-NTA affinity column.

Agarose gel electrophoresis results showed that a spectific band around 492 bp was amplified from *A.pernyi* cDNA as template (Figure 6a).The RT-PCR fragment was digested with *Bam*H I and *Hind* III, followed by subcloning into pET-28a(+) vector which was digested with the same restriction enzymes. The positive clone was confirmed by the double digestion and agarose gel analysis (Figure 6a). Sequencing analysis showed the ORF sequence cloned in the recombinant plasmid completely matched up with the sequence we excepted. SDS-PAGE analysis showed that the DAP3 recombined protein was expressed in *E.coli* cells with an approximate molecular weight of 21.6 kDa, which was consistent with the predicted molecular weight of the fusion protein HIS-DAP3 (Figure 6b). Next, the DAP3 fusion protein was highly purified with up to 92% purity, which was estimated by software BandScan V 5.0 (Figure 6b)

### SOD activity was detected in the HIS-DAP3 recombinant protein

**Figure 7:**
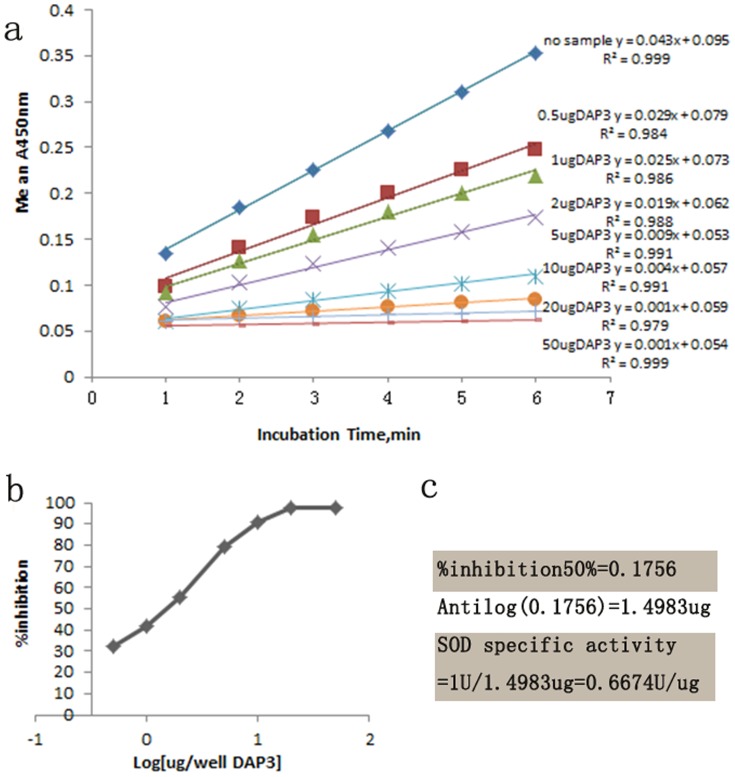
SOD activity detection of purified protein HIS-DAP3. (**a**) Change in absorbance at 450 nm with time for a DAP3 dilution series. A linear regression analysis of the reaction rates during the first 6 minutes of incubation is shown. (**b**) Inhibition curve for the DAP3 dilution series shown in figure 7-a. Results shown are the averages from three separate experiments. (**c**) Calculation of the SOD activity.

Because sequence analysis showed that a specific SOD domain located in the DAP3 protein, we want to know if DAP3 protein has SOD activity as predicted. The results of HT Superoxide Dismutase Assay showed that the absorbance values at 450 nm decreased while the concentration of DAP3 protein increased, indicating DAP3 protein possesses high SOD activity (Figure 7). The standard curve analysis showed that the SOD activity of DAP3 protein was 0.6674 U/µg.

### The expression level of DAP3 was regulated by oxidative stress response

**Figure 8:**
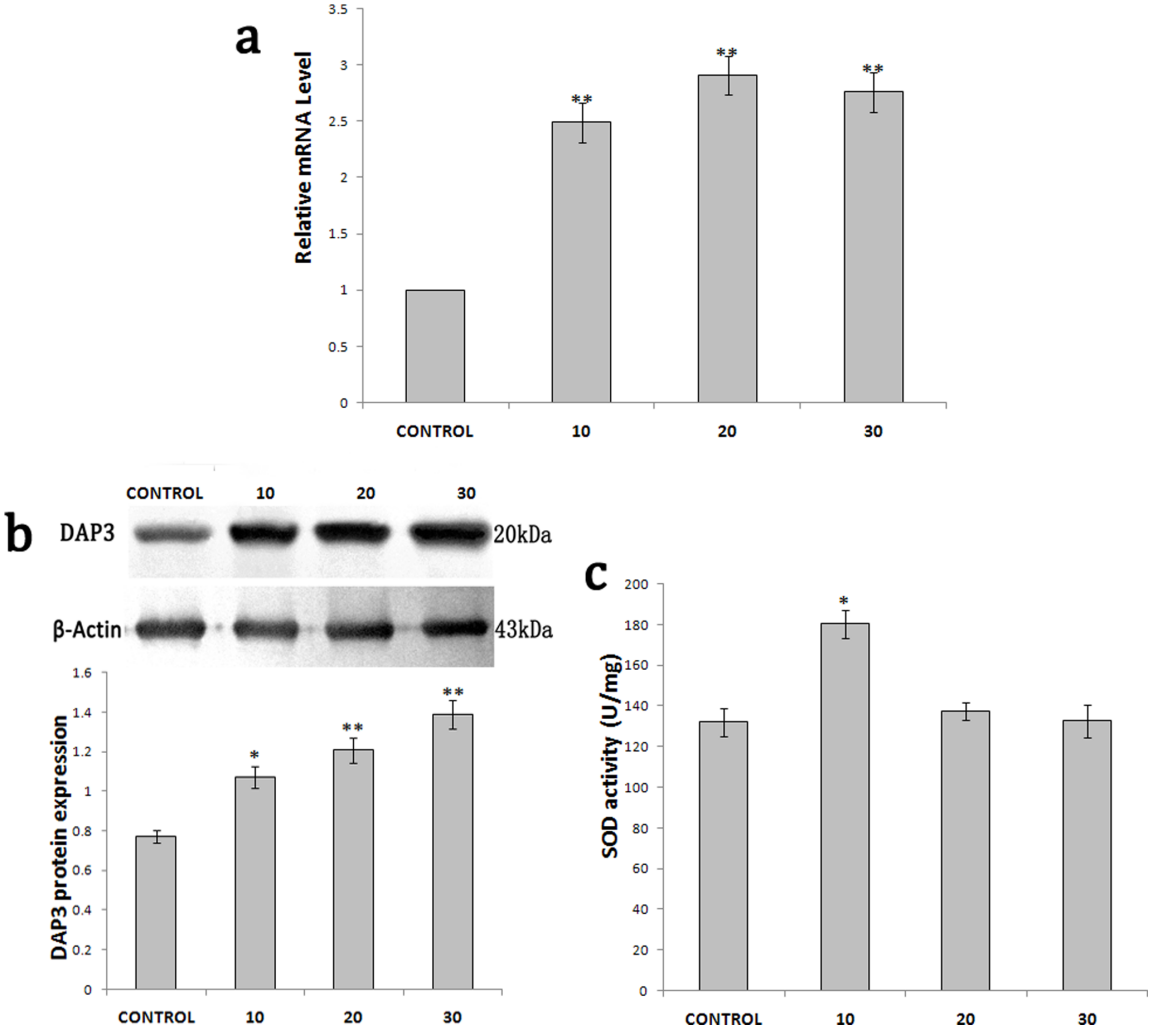
Effects of UV irradiation on the transcription and expression level and the SOD activity of DAP3 in *A.pernyi* pupae. (**a**) Relative mRNA levels of DAP3 were detected by qRT-PCR with β-actin as an internal control. Results are expressed as mean ± SEM of three independent experiments. **p<0.01 vs control. (**b**) Protein levels of DAP3 revealed by Western blot. *p<0.05 vs control, **p<0.01 vs control. (**c**) Effects of UV irradiation on SOD activity of *A.pernyi*pupae. * p<0.05 vs control.

Next, we want to know if the SOD activity of DAP3 was regulated by oxidative stress. As shown in Figure 8 a–b, the mRNA and protein level of DAP3 in the pupae began to increase at 10 min post UV light exposure and maintained a high level until 30 min, compared to the control group with no UV light exposure. On the other hand, the SOD activity in the lysate of *A.pernyi* pupae also significantly increased at 10 min post UV exposure (*p* = 0.001) and transiently returned to normal level at 20 and 30 min (Figure 8c), indicating that DAP3 might play a potential role in response to oxidative stress in *A.pernyi* pupae.

## Discussion

Previous studies showed that DAP proteins are abundant in diapause insect while little or no in the non-diapause insects [2], [3]. DAP is considered as a marker of the diapause of insect [5]. The analysis of different tissues of 5th instar larvae of *A.pernyi* showed high transcription level of DAP3 in the fat body (Figure 5a), while the protein is highly found in epidermis hemolymph, head andfat body (Figure 5b). The results suggest that DAP3 mRNA is mainly synthesized in fat body and DAP3 protein might be secreted into hemolymph, head and epidermis in the 5th instar *A.pernyi* larvae from the fat body. The finding is similar to the character of Cu/Zn-SOD, which secreted into hemolymph after synthesized in fat body [20], [21]. Moreover, we found that DAP3 is expressed in both diapause pupae and non-diapause pupae, which is not identical to the general characteristics of DAP proteins. In the diapause development stage, the mRNA and protein of DAP3 was found declined (Figure 4), which is consistent with the characteristics of DAP proteins reported previously [2], [3]. But in the *A.pernyi* larvae, especially in 1st and 5th instar larvae, DAP3 is also clearly detected. The results suggest that DAP3 might have distinct functions from the other DAP proteins identified before. It is speculated that DAP3 might take part in the termination of *A.pernyi* diapause, rather than the occurrence of diapause.

A problem should be pointed out that the enhancement of the mRNA level and protein expression level of DAP3 appeared to be not synchronous dramatically. It might arise from the different sensitivity of qRT-PCR and western blot methods. Furthermore, in Eukaryotic gene expression, the transcription and translation always have time and space intervals, which might contribute to the difference [22]. On the other side, it might be a smart protective mechanism used by the insects during evolution. The insects synthesize a large amount of mRNAs in response to the potential survival challenge in larval stage, which could be translated expressed into protein quickly in case of necessity and could also be degraded in other cases without waste of energy for dispensable protein production. The detailed reason remains to be further studied.

Studies in *Caenorhabditis elegans* showed that loss of extracellular Cu/Zn-SOD-4 enhances Daf-2 (insulin receptor) longevity and induces constitutive diapause [23], [24], [25]. Research aiming to the metabolism of H_2_O_2_ in *B.mori* revealed that the level of H_2_O_2_ was significant higher in the diapause eggs compared to non-diapause eggs, which suggests H_2_O_2_ may be involved in the termination of diapause eggs [26]. Previous studies showed that H_2_O_2_ could activate the release of diapause hormone (DH) and facilitate the progeny diapause decision by DH without the expression alteration of DH gene [27]. It is well known that H_2_O_2_ can be produced by SOD and degraded by catalase enzyme (CAT). Therefore, we speculate that SOD might play indirect roles in regulation of diapause by synthesis of high level of H_2_O_2_, just like TIME-EA4 in *B.mori* diapause egg.

Conserved domain of Cu/Zn-SOD was identified in DAP3 protein and the homology of DAP3 with Cu/Zn-SOD is highly conserved (Figure 1–2). We also found that DAP3 has high SOD activity (Figure 7). In consideration of the response to oxidative stress (Figure 8), the results suggest that DAP3 might be a novel protein of Cu/Zn-SOD, which might play potential roles in regulation of diapause in *A.pernyi*.
